# Simultaneous stereotactic radiosurgery of multiple brain metastases using single-isocenter dynamic conformal arc therapy: a prospective monocentric registry trial

**DOI:** 10.1007/s00066-021-01773-6

**Published:** 2021-04-21

**Authors:** Raphael Bodensohn, Anna-Lena Kaempfel, Daniel Felix Fleischmann, Indrawati Hadi, Jan Hofmaier, Sylvia Garny, Michael Reiner, Robert Forbrig, Stefanie Corradini, Niklas Thon, Claus Belka, Maximilian Niyazi

**Affiliations:** 1 Department of Radiation Oncology, University Hospital, LMU Munich, Marchioninistraße 15, 81377 Munich, Germany; 2 Institute of Neuroradiology, University Hospital, LMU Munich, Marchioninistraße 15, 81377 Munich, Germany; 3 Department of Neurosurgery, University Hospital, LMU Munich, Marchioninistraße 15, 81377 Munich, Germany; 4 German Cancer Consortium (DKTK), Munich, Germany; 5 German Cancer Research Center (DKFZ), Heidelberg, Germany

**Keywords:** Radiotherapy, Linear accelerator, Local ablative therapy, Oligometastases, Elements Multiple Brain Mets®

## Abstract

**Background:**

Single-isocenter dynamic conformal arc (SIDCA) therapy is a technically efficient way of delivering stereotactic radiosurgery (SRS) to multiple metastases simultaneously. This study reports on the safety and feasibility of linear accelerator (LINAC) based SRS with SIDCA for patients with multiple brain metastases.

**Methods:**

All patients who received SRS with this technique between November 2017 and June 2019 within a prospective registry trial were included. The patients were irradiated with a dedicated planning tool for multiple brain metastases using a LINAC with a 5 mm multileaf collimator. Follow-up was performed every 3 months, including clinical and radiological examination with cranial magnetic resonance imaging (MRI). These early data were analyzed using descriptive statistics and the Kaplan–Meier method.

**Results:**

A total of 65 patients with 254 lesions (range 2–12) were included in this analysis. Median beam-on time was 23 min. The median follow-up at the time of analysis was 13 months (95% CI 11.1–14.9). Median overall survival and median intracranial progression-free survival was 15 months (95% CI 7.7–22.3) and 7 months (95% CI 3.9–10.0), respectively. Intracranial and local control after 1 year was 64.6 and 97.5%, respectively. During follow-up, CTCAE grade I adverse effects (AE) were experienced by 29 patients (44.6%; 18 of them therapy related, 27.7%), CTCAE grade II AEs by four patients (6.2%; one of them therapy related, 1.5%), and CTCAE grade III by three patients (4.6%; none of them therapy related). Two lesions (0.8%) in two patients (3.1%) were histopathologically proven to be radiation necrosis.

**Conclusion:**

Simultaneous SRS using SIDCA seems to be a feasible and safe treatment for patients with multiple brain metastases.

## Introduction

Brain metastases (BM) occur in approximately 30% of all cancer patients [1]. Without treatment, BM are often associated with a dismal prognosis and a survival of only about 1 month [2]. Singular or solitary metastases are treated locally with stereotactic radiosurgery (SRS) or surgical resection, preferably in combination with adjuvant (stereotactic) radiotherapy [3–5]. Surgical resection is most often chosen in case of large and/or symptomatic lesions, because it can immediately relieve the patient from the burden of space-occupying effects.

However, almost 80% of all patients with BM have more than one metastasis [1, 2]. For two to four metastases of limited size, SRS is the first choice, as this procedure achieves a fast and effective treatment with high local tumor control and only rare side effects [6, 7]. For patients with multiple BMs, whole-brain radiotherapy (WBRT) is the mainstay of treatment [8]. WBRT is thought to delay neurological symptoms and prolong intracranial control; however, patients with poor performance at baseline are unlikely to benefit [9]. Moreover, WBRT is associated with a spectrum of toxicities that often result in irreversible cognitive decline even within a few months after treatment [10–12].

Through the improvement of systemic therapy with, e.g., immunotherapy targeting Programmed cell death protein 1 (PD‑1) or Programmed death-ligand 1 (PD‑L1), the survival of patients is steadily improving even in advanced cancer stages [13]. This also includes patients who have been treated with WBRT for multiple BMs. As a result, BM patients are now more frequently affected by a treatment-associated long-term neurocognitive decline; therefore, current approaches are trying to preserve cognitive function following WBRT [10, 11, 14–20]. The ongoing multicentric HIPPORAD (NOA-14) trial addresses this approach (results are pending) [16].

A different approach is the use of single-high-dose SRS for more than four BMs, while sparing the remaining brain parenchyma [6, 21]. Yamamoto et al. applied SRS to up to ten BMs in a multicentric prospective non-randomized observational study and showed that survival and toxicity for patients with five to ten BMs was non-inferior to patients with two to four BMs [22]. Even the long-term effects after a median follow-up of 12.0 months (range 0.3–67.5 months) were non-inferior [23]. According to further reports by Yamamoto et al., even more than ten BMs may be eligible for SRS in carefully selected patients [24, 25].

Although Yamamoto et al. have demonstrated non-inferiority, a major problem of SRS of multiple metastases is the cumulative duration of multiple treatments [26]. When using conventional multi-isocenter linear accelerator (LINAC) based SRS, plans get exponentially more complex with each additional metastasis, as contributions of one lesion’s dose to the other lesions have to be taken into account. Since each metastasis is treated with a separate isocenter, additional time is required for patient setup and verification. For three BMs, this can easily add up to treatment times of 60 min, as each metastasis often requires 20 min [27, 28]. Therefore, not only the treatment planning but also the irradiation itself is a time-consuming process.

To solve this problem, specific algorithms have been developed for Volumetric modulated arc therapy (VMAT) or dynamic arc therapy, in which a single isocenter is used while all metastases are simultaneously treated [29, 30]. Another approach to treat multiple metastases simultaneously is robotic radiosurgery using, for example, CyberKnife® (Accuray Incorporated, Sunnyvale, CA, USA), which however seems to be a bit more time-consuming than LINAC approaches [31]. Brainlab (Munich, Germany) has developed a specific single-isocenter 4π dynamic conformal arc (SIDCA) algorithm and included it in their Elements Multiple Brain Mets® SRS software. To the best of our knowledge, the Department of Radiation Oncology of the University Hospital, LMU Munich, was the first center worldwide to use this technique to irradiate multiple BMs simultaneously using a VersaHD® LINAC (Elekta, Stockholm, Sweden). Moreover, there are only a few reports of clinical experience with this new technique. In this study, early results of monocentric prospective data were evaluated with regard to safety and feasibility of SRS using SIDCA.

## Materials and methods

### Study design and participants

This study was designed as a prospective single-armed registry trial. In order to be included, a patient was required to have two or more brain metastases with a diameter of 3 to 25 mm each. With a margin of 1 mm added to the GTV, the smallest diameter of the planning target volume (PTV) was 5 mm, which corresponds to the isocentric width of one leaf of the VersaHD® multileaf collimator (Elekta, Stockholm, Sweden). All tumor entities except lymphoma, germinoma, or small-cell lung cancer were included. Patients with disseminated cerebral metastases or signs of leptomeningeal disease were excluded and received other treatments (such as WBRT) instead. Prior cranial radiotherapy was not an exclusion criterion; however, the treated metastases were not allowed to have been directly irradiated with local ablation (simultaneous boost during WBRT or prior SRS). This study was approved by the ethics committee of the LMU University Hospital Munich (nr. 573-15). Informed consent was obtained from all participants.

### Planning and prescription

In addition to a contrast-enhanced planning computed tomography (CT) scan (with iodine-based contrast agents), all patients received magnetic resonance imaging (MRI) scans with gadolinium-based contrast agents, both with a slice thickness of 1 mm. Planning CT and MRI were ideally not older than 1 week (maximum 2 weeks old) at the time of irradiation; usually both were done on the same day to reduce matching discrepancies. Patients were fixated with double-layer thermoplastic masks with headSTEP® from Innovative Technologie Völp (Innsbruck, Austria). Delineation and planning were accomplished using the Elements Multiple Brain Mets® SRS application by Brainlab (Munich, Germany). The dose prescription to each metastasis was dependent on its size and proximity to organs at risk (mainly optical pathway and brainstem), and was usually 18 Gy for metastases of 4.2–15.0 cm^3^ size and 20 Gy for <4.2 cm^3^ (prescribed to the 80% isodose). The prescription dose depending on the size is listed in Table 1. For large metastases close to critical structures, 15 Gy to the 80% isodose could be prescribed. Additionally, the number of targets was taken into account during dose prescription. Sahgal et al. presented a model in which the relative increase of the V12 value of the brain (brain volume which received 12 Gy) with a higher number of brain metastases was shown; he therefore recommended “a decrease in the prescription dose of the order of 1 Gy when five or more lesions are treated with SRS” [32]. Following this recommendation, the prescription dose of each target was usually decreased by 1 Gy once the number of metastases exceeded 5. For the PTV, the metastasis’ volume was isotropically expanded by a margin of 1 mm. Image guidance was achieved using ExacTrac® (Brainlab, Munich, Germany) which was connected to the HexaPOD® system (Elekta, Stockholm, Sweden) [33]. With this technique a deviation of the patients’ position smaller than 0.1 mm can be achieved.

**Table 1:** The prescribed dose depending on the tumor’s size

Volume of metastasis	Prescribed dose	Isodose
<4.2 cm^3^	20 Gy	80%
4.2–15.0 cm^3^	18 Gy	80%
>15.0 cm^3^	15 Gy	80%

### Prophylactic antiedematous treatment

For irradiation, a prophylactic dexamethasone scheme was administered which usually consisted of 4–2–0 mg on the day of irradiation, followed by 2–1–0 mg, 1–1–0 mg, and 1–0–0 mg within the first 3 days after treatment. The dosage was adjusted depending on the size or location of the metastases or the presence of edema and comorbidities (e.g., diabetes) requiring appropriate reduction. For prevention of gastric ulcers, pantoprazole 40 mg per day was prescribed during the days of dexamethasone treatment.

### Follow-up

To monitor intracranial local tumor control and the formation of new metastases, contrast-enhanced MRI with a slice thickness of 1 mm was performed every 3 months after treatment. Whenever new metastases were detected, further treatment with SRS, WBRT, or surgery was evaluated. In case of an increase in the size of the metastases after irradiation, differentiation between treatment-related image changes and tumor progression was performed using fluoroethyl-L-tyrosine positron-emission tomography (FET PET) [34]. In case of inconclusive findings of FET PET, a stereotactic biopsy was performed. In addition to monitoring intracranial control, the patients were clinically and neurologically examined during the follow-up visit. A change in treatment regimens or extracranial tumor control was recorded as well. The reported AE were described using the Common Terminology Criteria for Adverse Events (CTCAE) version 5.0.

### Statistics

Statistical analysis was performed using IBM SPSS Statistics version 22 (IBM, Armonk, New York, USA). Descriptive analysis was performed to describe patient and treatment characteristics. Survival analysis was performed using Kaplan–Meier estimators.

## Results

Overall, 65 patients, 35 (53.8%) male and 30 (46.2%) female, were treated with SIDCA. The first patient underwent SRS in November 2017 and the last patient in June 2019. The median age at the time of SRS was 61.5 years (range 22–85 years). With 40 (61.5%) patients, the most frequent entity was non-small-cell lung cancer (NSCLC), including 34 adenocarcinomas, 2 squamous cell carcinomas, and 4 neuroendocrine carcinomas, followed by melanoma with 10 (15.4%) patients and breast cancer with 7 (10.8%) patients. 25 (38.5%) patients had no extracranial metastases and 40 (61.5%) patients had extracranial metastases at the time of treatment. The median Karnofsky performance status at the time of radiosurgery was 90% (range 50–100%). The median follow-up at the time of data analysis was 13 months (95% CI: 11.1–14.9 months). 15 (23.7%) patients had received cranial irradiation before: 6 (9.2%) WBRT, 1 (1.5%) WBRT with simultaneous boost, 8 prior SRS (boost and SRS were applied to different lesions than those treated now). Details of the patient characteristics are listed in Table 2.

**Table 2:** Patient characteristics

Characteristic	Number of patients	Percentage (*N* = 65)
*Age (years) at first stereotactic radiosurgery (SRS): median 61.5 [range 22–85]*		
*Gender*		
Female	30	46.2%
Male	35	53.8%
*Karnofsky performance status (KPS) at first SRS (median 90)*		
50	1	1.5%
60	3	4.6%
70–80	25	38.5%
90–100	36	55.4%
*Graded prognostic assessment (GPA) score at first SRS (median 2)*		
1–1.5	16	24.6%
2–2.5	35	53.8%
3–3.5	11	16.9%
4	1	1.5%
Unknown	2	3.1%
*Recursive partitioning analysis (RPA) score at first SRS (median II)*		
I	22	33.80%
II	40	61.50%
III	3	4.60%
*Tumor entity of primary tumor*		
Adenocarcinoma lung	34	52.3%
Neuroendocrine carcinoma lung	4	6.2%
Squamous cell carcinoma lung	2	3.1%
Breast cancer	7	10.8%
Malignant melanoma	10	15.4%
Renal cell cancer	4	6.2%
Colon cancer	1	1.50%
Stomach cancer	1	1.50%
Uterine sarcoma	1	1.50%
Salivary gland carcinoma	1	1.50%
*Median follow up time 13 months (95% CI 11.1–14.9)*		
*Therapy following intracranial progression (N* *=* *18)*		
Second SRS	9	13.8%
Third SRS	1	1.5%
WBRT	8	12.3%
Best supportive care	2	1.5%
*Cerebral metastases per patient (median 3)*		
2	18	27.7%
3	19	29.2%
4	9	13.8%
5	9	13.8%
6	3	4.6%
7	2	3.1%
8	2	3.1%
9	0	0.0%
10	2	3.1%
11	0	0.0%
12	1	1.5%
*Immunotherapy/targeted therapy (38 patients)*		
Pembrolizumab	17	26.2%
Nivolumab	12	18.5%
Bevacizumab	4	6.2%
Durvalumab	2	3.1%
Atezolizumab	1	1.5%
Ramucirumab	1	1.5%
Nivolumab/Ipilimumab	6	9.2%
Trastuzumab/pertuzumab	1	1.5%
*Small molecules (14 patients)*		
Afatinib	4	6.2%
Osimertinib	3	4.6%
Axitinib	1	1.5%
Palbociclib	1	1.5%
Cabozantinib	1	1.5%
Sunitinib	1	1.5%
Dabrafenib/trametinib	5	7.7%
Binimetinib/encorafenib	1	1.5%
Crizotinib/alectinib	1	1.5%
Crizotinib/afatinib	1	1.5%

**Fig. 1:**
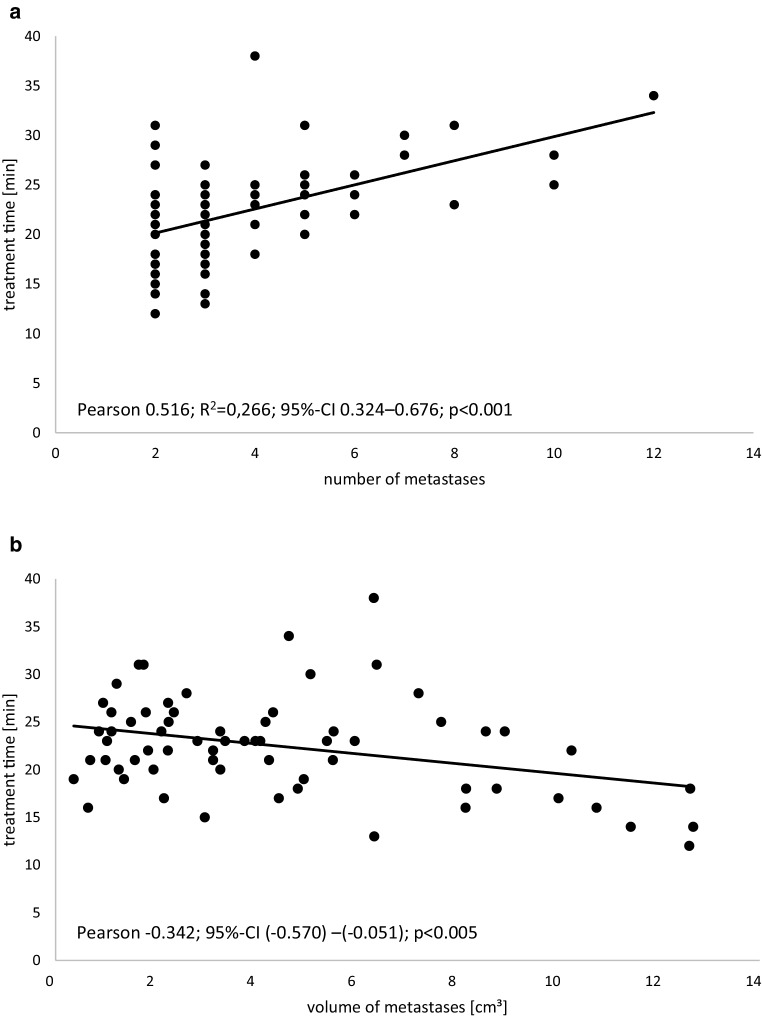
Pearson correlation of treatment time with total volume of metastases and number of metastases

Overall, 254 lesions were treated, of which two (0.8%) were located in the brainstem. The median number of treated lesions per patient per therapy session was three (range 2–12). The median treatment time of the simultaneous irradiation of all metastases at once was 23 min (range 12–38 min). There was a significant correlation between number of metastases and treatment time (Pearson 0.516; 95% CI 0.324–0.676; *p* < 0.001), and total volume of metastases and treatment time (Pearson: −0.342, 95% CI −0.570–−0.051, *p* < 0.005) which are depicted in Fig. 1. The median dose to each metastasis was 19 Gy (range 15–20 Gy). More information on the dose delivered to the organs at risk and to the irradiated metastases is given in Tables 3 and 4.

**Table 3:** Dosimetric and volumetric data of the irradiated metastases

Metastases (*n* = 254)	Median	Range
Volume GTV (cm^3^)	0.193	0.010–9.184
Volume PTV (cm^3^)	0.483	0.060–11.900
Cumulative volume PTV (cm^3^)	3.373	0.361–12.682
Median dose GTV (Gy)	21.99	16.56–25.04
Median dose PTV (Gy)	20.93	16.19–23.95
PTV prescription (Gy)	19	15–20

*GTV* gross tumor volume; *PTV* planning tumor volume

**Table 4:** Dosimetric and volumetric data of the organs at risk (OAR)

Organs at risk (*N* = 65)	Median mean dose (Gy)	Mean dose range (Gy)	Median maximal dose (Gy)	Maximal dose range (Gy)
Whole brain	1.38	0.56–2.78	24.34	19.41–27.15
Left hippocampus	1.13	0.26–4.83	1.42	0.30–17.31
Right hippocampus	1.56	0.12–6.03	1.43	0.19–24.92
Brainstem	1.01	0.24–2.28	2.28	0.63–18.15
Optic chiasm	1.08	0.17–4.98	1.37	0.19–8.73
Cochlea left	0.78	0.11–4.99	0.55	0.13–6.75
Cochlea right	0.93	0.08–7.33	0.70	0.08–11.27
Eye left	0.50	0.12–1.85	0.67	0.16–3.66
Eye right	0.53	0.09–2.73	0.77	0.12–8.75
Lens left	0.43	0.11–1.43	0.46	0.13–1.78
Lens right	0.47	0.10–2.28	0.44	0.10–2.71
Optic nerve left	0.80	0.14–4.17	0.88	0.18–6.15
Optic nerve right	0.83	0.12–3.85	0.96	0.14–11.86
Optic tract left	1.18	0.20–4.94	1.17	0.31–11.62
Optic tract right	1.18	0.16–4.91	1.22	0.19–15.71

In total, 32 (49.2%) patients experienced AEs. In the first month after treatment, seven (10.8%) patients experienced acute AEs CTCAE grade 1, such as fatigue, vertigo, or cephalgia, of which all could be related to the SRS. One patient (1.5%) who received irradiation of four melanoma metastases experienced seizures CTCAE grade 2 due to intralesional hemorrhage a week after treatment. Overall, no acute AEs CTCAE grade 3 were reported. During follow-up, 28 patients (43.1%) presented AEs: 22 patients (33.8%) experienced CTCAE grade 1 (11 of them therapy related, 16.9%), four patients (6.2%) experienced CTCAE grade 2 (one of them therapy related, 1.5%), and three patients (4.6%) CTCAE grade 3 (none of them therapy related). The AEs are listed in detail in Table 5.

**Table 5:** Adverse events experienced by the patients

		CTCAE grade I	CTCAE grade II	CTCAE grade III
*Fatigue* (number of patients)		*26 [40.0%]*	–	–
*Fatigue* (number of events)	Acute	3 [4.6%]	–	–
	After 3 months	10 [15.4%]	–	–
	After 6 months	9 [13.8%]	–	–
	After 9 months	7 [10.8%]	–	–
	After 12 months	6 [9.2%]	–	–
	After 15 months	5 [7.7%]	–	–
	After 18 months	3 [4.6%]	–	–
*Cephalgia* (number of patients)		*8 [12.3%]*	*1 [1.5%]*	–
*Cephalgia* (number of events)	Acute	2 [3.1%]	–	–
	After 3 months	3 [4.6%]	–	–
	After 6 months	4 [6.2%]	1 [1.5%]	–
	After 9 months	4 [6.2%]	–	–
	After 12 months	1 [1.5%]	–	–
	After 15 months	1 [1.5%]	–	–
*Vertigo* (number of patients)		*10 [15.4%]*	–	–
*Vertigo* (number of events)	Acute	6 [9.2%]	–	–
	After 6 months	4 [6.2%]	–	–
	After 9 months	2 [3.1%]	–	–
	After 12 months	1 [1.5%]	–	–
	After 15 months	2 [3.1%]	–	–
	After 18 months	1 [1.5%]	–	–
*Motoric impairment* (number of patients)		*1 [1.5%]*	–	–
*Motoric impairment* (number of events)	After 3 months	1 [1.5%]	–	–
	After 9 months	1 [1.5%]	–	–
*Sensitivity disorder* (number of patients)		*2 [3.1%]*	–	–
*Sensitivity disorder* (number of events)	After 6 months	2 [3.1%]	–	–
	After 9 months	1 [1.5%]	–	–
*Aphasia* (number of patients)		*1 [1.5%]*	–	–
*Aphasia* (number of events)	Acute event	1 [1.5%]	–	–
	After 6 months	1 [1.5%]	–	–
*Neurocognitive impairment* (number of patients)		*18 [27.7%]*	–	–
*Neurocognitive impairment* (number of events)	Acute	2 [3.1%]	–	–
	After 3 months	4 [6.2%]	–	–
	After 6 months	1 [1.5%]	–	–
	After 9 months	8 [12.3%]	–	–
	After 12 months	3 [4.6%]	–	–
	After 15 months	3 [4.6%]	–	–
	Atfer 18 months	2 [3.1%]	–	–
*Seizure* (number of patients)		*4 [6.2%]*	–	*3 [4.6%]*
*Seizure* (number of events)	Acute event	–	1 [1.5%]	–
	After 3 months	1 [1.5%]	1 [1.5%]	2 [3.1%]
	After 6 months	1 [1.5%]	–	–
	After 9 months	–	–	1 [1.5%]
*Alopecia* (number of patients)		*7 [10.8%]*	*2 [3.1%]*	–
*Alopecia* (number of events)	After 3 months	4 [6.2%]	2 [3.1%]	–
	After 6 months	2 [3.1%]	–	–
	After 9 months	1 [1.5%]	–	–
	After 12 months	1 [1.5%]	–	–
	After 15 months	1 [1.5%]	–	–
*Radiation necrosis* (number of symptomatic patients)		–	–	–
Paresis left arm	After 1 month	1 [1.5%]	–	–
Vertigo	After 3 months	1 [1.5%]	–	–
Vertigo, nystagmus	After 6 months	1 [1.5%]	–	–

**Fig. 2:**
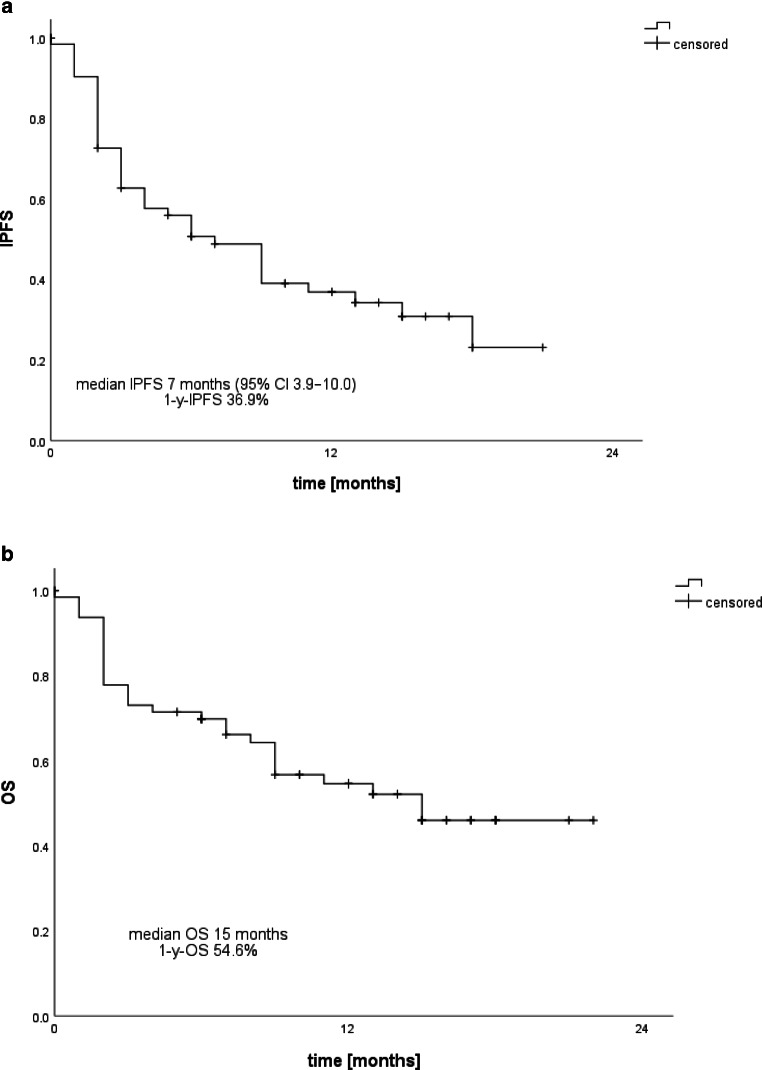
Kaplan–Meier curves of intracranial progression-free survival (iPFS) and overall survival (OS)

By December 2019, 30 patients (46.2%) had died, five of them with cerebral progressive disease. Most of the patients (26.2%) died within the first 3 months after radiosurgery. Detailed information is given in Fig. 2. Two patients were lost to follow-up. The median overall survival (OS) was 15 months (95% CI 7.7–22.3). OS hardly correlated with the calculated GPA score (Pearson 0.186; 95% CI −0.138–0.488; *p* = 0.145). There was no significant correlation between number of metastases and overall survival (Pearson: 0.132; 95% CI −0.114–0.366; *p* = 0.293). In the follow-up period, 36 patients (55.4%) had neither a malignant progressive lesion nor new metastases in the brain after radiosurgery. Local control of the irradiated lesions after 6 months and 1 year was 97.5%.

A total of 13 lesions (5.1%) of ten patients (16.9%) were progressive in size after treatment. Three lesions were diagnosed with radiation necrosis, one with tumor progression, and nine were classified clinically and neuroradiologically as pseudo-progression: these lesions will be highlighted in this paragraph. One lesion (0.4%) of a triple-negative breast cancer irradiated with 15 Gy had histopathologically verified tumor progression 6 months after radiosurgery. This patient additionally had multiple new metastases 1 year after therapy and was then treated with WBRT (30 Gy in 10 fractions). 21 months after SRS and 6 months after WBRT, the patient developed three new metastases: the pre-irradiated progressive lesion was surgically resected and the rest were treated with further SRS treatment. Symptomatic radiation necrosis (RN) was diagnosed in three patients (4.6%), of which two lesions (0.8%) in two patients (3.1%) were histopathologically verified. One of them was treated with steroids due to symptoms such as vertigo CTCAE grade 2 with nystagmus, which were almost identical to the initial symptoms. The other patient experienced vertigo and cephalgia CTCAE grade 1 but did not require any antiedematous treatment. Another patient developed symptomatic RN in two metastases, which was clinically and radiologically diagnosed, but no biopsy was performed. Both lesions were irradiated with 20 Gy. The patient suffered from paresis of the left arm and was first treated with dexamethasone and then with bevacizumab in analogy to Levin et al. after insufficient response [35, 36]. Nine lesions (3.5%) in seven patients (10.8%) were interpreted as pseudo-progression by an experienced neuroradiologist.

Moreover, 18 patients (27.7%) developed new brain metastases after their first radiosurgery during the follow-up period. After the first intracranial progression, eight patients received WBRT and eight patients received a second series of SRS. Two patients also received a third radiation series: one of them was treated with SRS after WBRT and the other one was treated with a third SRS course. Two patients are still under active surveillance after showing only limited progression. The median intracranial progression-free survival (iPFS) was 7 months (95% CI 3.9–10.0). The intracranial control rate was 73.0% after 6 months and 64.6% after 12 months.

A total of 38 patients (58.5%) were treated with immunotherapy at some point during the course of their disease. Twelve patients (18.5%) received immunotherapy within a time period of 4 weeks before and after treatment. Fourteen patients (21.5%) received tyrosine kinase inhibitors (TKI). Four of these patients (6.2%) were treated throughout the course of disease, during and after radiotherapy. Overall, these patients had only few AE, most of which were unrelated to the cranial irradiation (such as thyroiditis in one patient, or alopecia in two): two patients (3.1%) experienced cephalgia CTCAE grade I and two patients (3.1%) fatigue CTCAE grade I. However, one patient with malignant melanoma who received nivolumab until shortly before radiotherapy suffered from a cranial hemorrhage in the irradiated metastases a week after radiotherapy and died a month later. The symptoms included aphasia CTCAE grade I and seizure CTCAE grade II. No RN was detected among the patients who received immunotherapy at the time of radiation. Detailed information about immunotherapy and small molecule treatment is listed in Table 2.

## Discussion

To date, SRS has mostly been limited to patients with a certain number of metastases [6, 21]. The question remains whether there is a limit to how many metastases can be irradiated using SRS. Hatiboglu et al. suggested that the total tumor volume has a larger influence on the whole-brain dose than the number of metastases [37]. In fact, a dosimetric analysis by Becker et al. showed that theoretically, 40 metastases of different sizes could be irradiated radiosurgically with Gamma Knife® (Elekta, Stockholm, Sweden) before a whole-brain dose of 8 Gy, corresponding to the dose of a single fraction of WBRT, had been reached [38, 39]. As mentioned above, Yamamoto et al. were able to show that SRS of five to ten metastases is non-inferior to SRS of two to four metastases [22]. Treatment of more than four metastases might still be a reasonable approach; this is also recommended as an option in the NCCN guidelines for viable cases (version 1.2020). However, it is important to find a suitable radiation technique for this treatment.

This study tested the simultaneous irradiation of multiple metastases using SIDCA on a VersaHD® LINAC from Elekta (Stockholm, Sweden) by analyzing early results of a prospective cohort. Other techniques such as VMAT using HyperArc® (Varian Medical Systems, Palo Alto, California, USA) have been tested before with good results [30, 40, 41]. From a pure dosimetric point of view, SIDCA was compared with VMAT in a 2019 study: the SIDCA plans were reevaluated using Monaco® (Elekta, Stockholm, Sweden) as VMAT plans. According to the study, SIDCA can potentially ensure better protection of healthy brain tissue and superior treatment efficiency with a steeper dose gradient if the target volume is nearly spherical, while for non-spherical volumes VMAT was able to achieve higher conformity [42]. The SIDCA plans showed good dose coverage as they were optimized in such a way that no leaves are open between two metastases and no sequencing is present. So even with a leaf width of 5 mm, sufficient conformality was achieved.

In this study 29.2% of the treated patients had five or more metastases, which matches to MRI observations describing only about 50% with three or more metastases [43]. The limited number of patients with five or more metastases can be counted as a limitation of this study, as a larger number of patients with a higher number of metastases potentially could have more side effects or radiation necrosis and therefore shorter OS. In the cohort, however, there was no correlation between OS and the number of treated metastases, but a correlation might still have been seen if more patients with a larger number of metastases had been included. Therefore, this study alone cannot sufficiently show that SRS is feasible regardless of the number of metastases. But as other studies, such as Yamamoto et al., have shown non-inferiority regarding the outcome of patients with five or more metastases compared to patients with two to four metastases, the results of this study can be seen as analogous [22, 25]. The main goal of this study is, in the first place, to show that SRS with SIDCA is feasible and comparable to other techniques. Despite its limitations, the study’s results showed that SIDCA is a feasible approach for SRS of multiple metastases.

There is another recent study which evaluated single-isocenter stereotactic radiosurgery for multiple metastases with similar good results [45]. However, Palmer et al. used VMAT instead of dynamic conformal arc therapy, and applied the dose in 1–5 fractions instead of in a single fraction. In the latter study, 15-month (95% CI 7.7–22.3) OS was high compared to other similar studies: Yamamoto et al. had 10.8 months (05% CI 9.4–12.4) for patients with two to four and 10.8 months (9.1–12.7) for five to ten metastases [22]; Limon et al. had 5.8 months (95% CI 4.9–6.6) [44]; Palmer et al. had 13.2 months (95% CI 8.5–18.7 months) [45]. In a 2019 meta-analysis by Lehrer et al. including 24 trials with patients with single- and multi-fractioned SRT in definitive and postoperative settings, OS was median 7.9 months for patients who received single-fraction definitive SRS [46]. The reason for the better OS in this study compared to older cohorts is the advance of targeted therapy such as PD-L1-inhibitors in recent years, which has improved OS drastically in many settings [47–49]. This was probably also the reason for little correlation of the OS with the survival estimated by the GPA score.

As described in the results, the AEs were very limited, most of them only being CTCAE grade 1. Three patients experienced seizures CTCAE grade 3: one resulted from intracranial bleeding after a massive progression, another from leptomeningeal disease, and the last one occurred after receiving WBRT due to disseminated intracranial progress. Furthermore, five patients (7.6%) experienced CTCAE grade 2 AEs, one of which was cephalgia due to posttherapeutic edema, which could be treated with dexamethasone. One patient experienced seizures due to improper intake of anticonvulsive medication. Another one due to intracranial hemorrhage a week after SRS. The last two patients had alopecia following systemic treatment. With only two patients (3.1%) with treatment-related AEs CTCAE grade 2 and 3 each, the results are comparable to other literature: Palmer et al. (using VMAT with 1–5 fractions) only described radionecrosis of the lesions (*N* = 1014) at 1.4% and 0.9% for grade 2 and grade 3, respectively. Under the assumption that each lesion was in a separate patient (*N* = 173), this represented 0.1% and 5.2% for grade 2 and grade 3, respectively. Yamamoto et al. had around 7% grade 1 and 2 toxicities, 2% grade 3, 1% grade 4, and 1% grade 5 [22]. Unfortunately, other similar trials did not describe AEs in enough detail for better comparison, partly due to short follow-up [40].

Another advantage of the single-isocenter approach is the time-saving aspect: while multi-isocenter approaches need approximately 20 min per lesion, about the same time is needed for all lesions simultaneously with SIDCA (in this study median 23 min) [27, 28]. The simultaneous single-fraction approach also has a great advantage compared to the hypofractionated approach when combining SRS with systemic treatments, as SRS can more easily be applied between cycles.

Concerning RN, the incidence with four lesions (1.6%) in three patients (4.6%) was lower compared to other literature. In the meta-analysis by Lehrer et al., the rate of radiation necrosis was by 18.2% for single-fraction definitive SRS [46]. This can probably be explained by the small margin of 1 mm and the rationally adapted median dose to the PTVs of 19 Gy. Additionally, the limitation of having a limited number of patients with five or more metastases might also have played a role in this favorable number. The prophylactic administration of dexamethasone might have been a protective factor as well. Altogether, simultaneous SRS with SIDCA showed only few side effects in this prospective monocentric registry trial.

The only metastasis which had histologically verified tumor progression was a triple-negative breast cancer metastasis irradiated with merely 15 Gy (prescribed to 80% isodose) due to its size and closeness to the brainstem. Only two metastases in the cohort were irradiated with 15 Gy, of which one was not locally controlled. This could be an indication that a single-fraction dose of 15 Gy is not sufficient for local control. Other than that, local control was very high compared to data of other studies (97.5%): Palmer et al. had a 1-year local control of 99.0% [45]; Yamamoto et al. had 93.0% and 93.5% for patients with two to four and five to ten metastases, respectively [23]; in Lehrer et al., local control of the patients who received definitive single-fraction SRS was at 76.7% after 1 year [46].

Unfortunately, the number of patients receiving parallel immunotherapy was too low and their follow-up too short to show any significant additive effect, higher toxicities, or abscopal effects. This aspect will be highlighted further in the future.

## Conclusion

The simultaneous radiosurgical irradiation of multiple brain metastases using SIDCA could be applied safely and efficiently in this prospective monocentric registry trial with good local control and few treatment-related adverse effects or radiation necroses. Long-term observations for patients with over four metastases, especially in combination with systemic treatment, are needed for further validation of this finding.
